# Supplementary material for: Stakeholder perspectives on contributors to delayed and inaccurate diagnosis of cardiovascular disease and their implications for digital health technologies: a UK-based qualitative study
Source: BMJ Open. 2024 May 20;14(5):e080445. doi: 10.1136/bmjopen-2023-080445 (PMC11110589; doi:10.1136/bmjopen-2023-080445)
Supplement: Supplementary data [file bmjopen-2023-080445supp004.pdf]

````
title: "Supplemental Material - R Code"
output: html_document
date: "2023-09-13"
---

```{r setup, include=FALSE}
knitr::opts_chunk$set(echo = TRUE)

library(ggraph)
library(igraph)
library(tidyverse)
library(RColorBrewer)
library(ggplot2)
library(viridis)
library(readxl)
theme_set(theme_void())


library(treemap)

```

```{r read the data in}

d2 <- readr::read_csv(here::here("data/dendo_data_d2.csv"))
d1 <- readr::read_csv(here::here("data/dendo_data_d1.csv"))
d3 <- readr::read_csv(here::here("data/dendo_data_d3.csv"))

d1=data.frame(d1)
d2=data.frame(d2)
edges=rbind(d1,d2)

```

```{r}

# create a vertices data frame. One line per object of our hierarchy
vertices = data.frame(
  name = unique(c(as.character(edges$from), as.character(edges$to))) ,
  value = d3
)
# add a column with the group of each name.
vertices$group = edges$from[ match( vertices$name, edges$to ) ]
```

```{r}
vertices$id=NA
````
````

BMJ Publishing Group Limited (BMJ) disclaims all liability and responsibility arising from any reliance placed on this supplemental material which has been supplied by the author(s)


~~~
myleaves=which(is.na( match(vertices$name, edges$from) ))
nleaves=length(myleaves)
vertices$id[ myleaves ] = seq(1:nleaves)
vertices$angle= 90 - 360 * vertices$id / nleaves
vertices$angle= 0

# calculate the alignment of labels: right or left
# If I am on the left part of the plot, my labels have currently an angle < -90
vertices$hjust<-ifelse( vertices$angle < -90, 1, 0)
vertices$hjust<-ifelse(vertices$id > 10 & vertices$id < 24, 1, 0)
# flip angle BY to make them readable
vertices$angle<-ifelse(vertices$angle < -90, vertices$angle+180, vertices$angle)


```

```{r}

# Create a graph object
mygraph <- graph_from_data_frame(edges, vertices=vertices)

# prepare color
#mycolor <- colormap(colormap = colormaps$viridis, nshades = 6, format = "hex", alpha = 1,
reverse = FALSE)[sample(c(1:6), 10, replace=TRUE)]

# Make the plot
graph <- ggraph(mygraph, layout = 'dendrogram', circular = TRUE) +
  geom_edge_diagonal(colour="dark grey") +
  scale_edge_colour_distiller(palette = "RdPu") +
  #geom_node_text(aes(x = x*1.10, y=y*1.20, filter = leaf, label=name, angle = angle,
hjust=hjust, colour=group), size=3.5, alpha=1) +
  #geom_node_text(aes(x = x*1.15, y=y*1.15, filter = leaf, label=name, angle = angle,
hjust=hjust, colour=group), size=3.5, alpha=1) +
  geom_node_point(aes(filter = leaf, x = x*1.03, y=y*1.03, colour=group, size=value,
alpha=5)) +
  #geom_node_point(aes(filter = leaf, x = x*1.07, y=y*1.07, colour=group, size=value,
alpha=0.2)) +
  #scale_colour_manual(values= mycolor) +
  #scale_size_continuous( range = c(1,10) ) +
  theme_void() +
  theme(
    legend.position="none",
    plot.margin=unit(c(0,0,0,0),"cm"),
  ) +
  expand_limits(x = c(-0.5, 0.5), y = c(-0.5, 0.5))
graph
~~~

````
```
```{r}
compare_data <- readr::read_csv(here::here("data/tree_compare_data.csv"))


totalmap <- treemap(compare_data,
            index=c("theme","group"),
            vSize="size",
            type="index",
            palette= "Pastel2",
            fontcolor.labels=c("black", "white"),
            border.col = c("black", "white"),
             border.lwds=c(1,2),
            align.labels=list(
              c("left", "top"),
              c("right", "bottom")
              ),
           # inflate.labels=F,
           title = " ",
            #fontsize.title=12
            )
```
````
````
